# A collaborative learning approach to improving health worker performance in adolescent sexual and reproductive health service provision: a descriptive feasibility study in six health zones in the Democratic Republic of the Congo

**DOI:** 10.1080/16549716.2021.1985228

**Published:** 2021-10-31

**Authors:** Sylvie Olela Odimba, Frances Squires, Erin Ferenchick, Symplice Mbola Mbassi, Paul Chick, Marina Plesons, Venkatraman Chandra-Mouli

**Affiliations:** a Programme National de Lutte contre le VIH/SIDA, Ministry of Health, Kinshasa, Democratic Republic of the Congo; b Programme National de Lutte contre le VIH/SIDA, Catholic Organization for Relief and Development Aid (Cordaid), Kinshasa, Democratic Republic of the Congo; c Independent Expert; d Technical Advice and Partnership Department, The Global Fund to Fight Aids, Tuberculosis and Malaria, Geneva, Switzerland; e Universal Health Coverage /Life Course Cluster World Health Organization Regional Office for Africa Brazzaville, Republic of Congo; f Nigeria CT / C19 Funds, The Global Fund to Fight Aids, Tuberculosis and Malaria, Geneva, Switzerland; g Department of Sexual and Reproductive Health and Research, World Health Organization/ UNDP/UNFPA/UNICEF/WHO/World Bank Human Reproductive Programme

**Keywords:** Collaborative learning, adolescent, adolescent health services, health service provider performance, sexual and reproductive health

## Abstract

Poor performance among health service providers is a key barrier to high quality, adolescent-responsive health services. Collaborative learning has been shown to strengthen health service provider performance, but few studies have examined its implementation in adolescent health services. In this paper, we describe a collaborative learning approach for adolescent health service providers implemented as part of a project aiming to prevent HIV in adolescent girls and young women in the Democratic Republic of the Congo (DRC) and explore its feasibility, acceptability, benefits and challenges. To do so, we reviewed plans, budgets and progress reports, as well as nested implementation research related to the project. We also carried out a quantitative analysis of the number, location, participants and topics of collaborative learning sessions conducted as part of this initiative, and thematic analysis to synthesise findings on perceived benefits and challenges. Under the project, 32 collaborative learning sessions of approximately two-hour duration were held with up to 15 participants, most of whom were nurses, between June 2018 and May 2019. The project cost was approximately USD 135,000 over three years. Reported benefits included improving health service provider attitudes, knowledge and skills; ensuring delivery of non-judgemental, empathic and confidential health services; and improving communication and collaboration between health services and adolescents together with their parents. While the novelty of the approach in this context presented challenges, our results suggest that collaborative learning in adolescent health services is feasible, acceptable, and inexpensive. It may help strengthen the knowledge and skills of health service providers, build positive attitudes and motivation, and improve their performance and thereby the adolescent-responsiveness of health services. Further research is needed to confirm these results in other settings and to examine the impact of collaborative learning on the acceptability and uptake of health services.

## Background

Globally, adolescence is often viewed as a time of opportunity and relatively good health. However, adolescents can experience a range of health problems, including sexual and reproductive health (SRH) challenges [1,2]. In many contexts, lack of access to high quality and adolescent-responsive health services exacerbates the vulnerability of young people to poor SRH. In particular, poor clinical and interpersonal performance of health service providers is known to affect the provision and utilisation of SRH services for adolescents [3–5]. In this paper, we describe and analyse a collaborative learning approach being implemented with the aim of improving health service provider capacity, motivation and performance within adolescent SRH health services in the Democratic Republic of the Congo (DRC).

Collaborative learning is an approach to teaching and learning where groups of learners work together to identify and discuss issues and challenges, seek appropriate solutions, plan for future actions, and advance their individual and collective learning goals [6]. It draws on a cluster of inter-related social and behavioural science theories that aim to describe how practices, norms and behaviours become embedded in different social settings (including health services), such as Social Network Theory [7], Diffusion of Innovations Theory [8,9], and Normalization Process Theory [10]. Group problem solving and collaborative learning have been shown to be effective in improving the attitudes and performance of health service providers in low- and middle-income countries [11–13]. However, few studies have assessed the feasibility and acceptability of collaborative learning in the context of adolescent health; further, the most comprehensive of these studies was conducted in a European setting [14].

In this paper, we describe the characteristics of a collaborative learning approach for health service providers working in adolescent health being implemented in six health zones in two provinces of the DRC (Kasai Oriental and Kinshasa). We also aim to explore its feasibility and acceptability and to examine preliminary evidence of its benefits and challenges.

### Context

In the DRC, adolescents are at risk of poor health outcomes linked to the socio-economic and environmental context. This large country in central Africa faces complex challenges including poverty, conflicts, a history of state fragility, and public health issues – including both infectious diseases such as Ebola outbreaks [15] and a growing burden of non-communicable diseases such as stroke and ischaemic heart disease [16]. While there are efforts to strengthen the DRC health system, ongoing challenges include weak governance and accountability and inadequate government funding; fragmented external aid; shortages and uneven distribution of health care workers, shortages of essential supplies; low education standards, motivation, and performance among health care workers; and low affordability, high out-of-pocket payments and poor access in the rural areas [17]. Furthermore, individual factors (including lack of knowledge and understanding about health issues including SRH) combine with poor access to accurate information and services and social factors (including poverty, pressure to have an early sexual debut and to engage in unprotected sexual activity including in the context of transactional and coerced sex) to increase the likelihood of SRH problems [18].

Rates of early sexual initiation and childbearing, within and outside marriage, are high in the DRC. The majority of girls have had sexual intercourse by the age of 19 years [18.] Over a third of them (37%) have been married by the age of 18 years [19]. These factors contribute to an adolescent birth rate of 138/1000 among girls aged 15–19 years [20], which in turn contributes to high levels of maternal mortality and morbidity and unsafe abortion. There are an estimated 5100 new HIV infections among young people aged 15–24 years each year; the vast majority are among young women [21]. As in other parts of Sub-Saharan Africa, it is likely that sexual and other forms of violence, transactional sex, and sex work are a significant risk factor for HIV infection amongst young women in the DRC [22]. The maternal mortality rate is unacceptably high at an estimated 473 per 100,000 live births in 2017 [23]. Physical and sexual violence is highly prevalent in the DRC, with 51% of women experiencing physical or sexual intimate partner violence in their lifetime [24] and 16% of young women experiencing sexual violence by the age of 19 years [25].

To address these issues, a multi-sectoral, contextualised intervention package, including community-based, school-based and health facility-based interventions, was piloted in the DRC [17]. The interventions were funded by The Global Fund to Fight AIDS, Tuberculosis and Malaria (Global Fund) and supported by the Ministry of Health through two programmes: the ‘Programme national de lutte contre le SIDA’ (PNLS) and the ‘Programme national de la santé des adolescents’ (PNSA). The Catholic Organization for Relief and Development Aid (Cordaid) served as the primary recipient of the Global Fund grant, and the Réseau National des ONG pour le Développement de la Femme (RENADEF) served as the sub-recipient to facilitate implementation at the operational level. Three health zones in Kinshasa and three in Kasai Oriental were selected to participate in this project on the basis of their high HIV prevalence among young women aged 15–24 years, and the presence of health services already supported by the Global Fund through existing grants. Technical support on the health facility component of the project was provided by the World Health Organization (WHO). The overall approach had three main objectives:

1. To increase the proportion of adolescent girls and young women with adequate knowledge and skills of SRH, HIV, human rights, and gender-based violence;
2. To reduce the proportion of adolescent girls and young women who have been victims of gender-based violence in the school environment;
3. To improve access to and the delivery of quality adolescent-responsive health services, where adolescents could obtain the SRH services they need.

Annex 1 contains a logical model for the Project titled Preventing HIV in adolescent girls and young women in the Democratic Republic of Congo. It sets out the health outcomes, the adolescent behaviours most directly related to the health outcomes, the determinants of adolescents’ behaviours, the interventions to address the determinants and the intervention support activities.

The collaborative learning approach described in this paper was implemented under the third objective of the package. It was designed to address an identified need to move beyond the traditional didactic training model and complement training with more innovative approaches for adult learning. The Global Fund and WHO worked with the government and implementing partners to adapt and implement an approach that had been applied elsewhere, in the context of the DRC.

The collaborative learning approach was delivered as part of a planned package of interventions aiming to improve health service provider capacity, motivation, and performance, and to stimulate problem identification and solving. The package included job descriptions, training, refresher training, desk-reference tools, and collaborative learning (Supportive supervision was meant to be part of the package but it was implemented). With the technical support of WHO, two initial two-day training sessions were held in late 2017 and early 2018 for 50 health service providers focusing on developing a shared understanding of adolescence, key issues affecting adolescents and their development, adolescents’ specific health needs, and how to respond to their SRH information and service needs with empathy and sensitivity. In March 2018, a four-day meeting was held with the Ministry of Health, specifically PNLS and PNSA, implementing partners, members of the steering committee responsible for the project implementation and key stakeholders to the collaborative learning approach. This meeting was used to validate and finalise the ‘Collaborative Learning Guide for Improving the Performance of Health Service Providers,’ which was developed by the WHO in collaboration with the Ministry of Health and implementing partners specifically for use in the DRC, based on experience from a separate collaborative learning approach implemented in Moldova [14,26].

In April 2018, the Ministry of Health, Cordaid and the Global Fund, with technical support from WHO, organised a five-day workshop to train 30 facilitators from all levels of the health system on the collaborative learning approach. The facilitators were purposefully selected by the project manager in discussion with senior clinical staff (usually the senior medical officer) in each health zone. All facilitators were directly involved in providing health services to adolescents in their health zone. The training was an opportunity to strengthen the capacity of facilitators on topics central to adolescent health, such as unintended and early pregnancy, unsafe abortion, contraception and family planning, HIV/AIDS and sexually transmitted infections, violence and injuries, mental health, substance abuse, nutrition, prevention of cervical cancer, and the provision of adolescent-responsive health services. In addition, key written resources including the WHO Adolescent Job Aid [27], relevant national strategies, and quality standards were disseminated to support provision of quality services to adolescents.

After participating in the training, facilitators were responsible for training health service providers and managers from the participating health services. Monitoring support was provided by key staff from the Ministry of Health, Cordaid, and RENADEF. The WHO Regional Office for Africa also provided monitoring support, including through visits in September 2018 and March 2019 to review progress in implementing the collaborative learning approach, provide ongoing capacity building to the facilitators, and agree on next steps to strengthen implementation.

## Methods

We collected data from a variety of sources.

- First, we reviewed plans, budgets and progress reports related to the project titled Preventing HIV in adolescent girls and young women in the Democratic Republic of Congo and the nested implementation research titled Implementation research to test the feasibility, acceptability and effectiveness of proven approaches in improving health worker performance on adolescent sexual and reproductive health, in the context of a GFATM-supported project in the Democratic Republic of Congo.
- Second, we drew heavily from the collaborative learning session reports, designed as a monitoring tool for the project, which were used to collect information on the sessions, including the topics discussed, participants, steps identified to resolve problems, and recommendations to be implemented. These session reports were prepared by facilitators using a template that was developed by the WHO and the Global Fund and validated by PNLS and PNSA.
- Third, we reviewed the monthly reports of the health zones, which incorporated the individual collaborative learning session reports and added the perspectives of health facility managers.
- Fourth, we gathered further details on the implementation of the collaborative learning approach from two monitoring mission reports. These missions were conducted by a team made up of PNLS and PNSA focal points, representatives of WHO, Cordaid, and RENADEF, in September 2018 and February 2019.
- Finally, we examined budget documents to collate available information on the costs of the collaborative learning approach.

The documents gathered were reviewed by the authors to conduct a quantitative analysis of the number, location, participants and topics of the sessions. They also conducted thematic analysis by joint document review and discussion to synthesise findings on the session topics, implementation challenges, and perceived benefits.

## Results

### Characteristics of the collaborative learning approach

Collaborative learning sessions were started in June 2018 in all six health zones. Between June 2018 and May 2019, a total of 32 sessions were held. At each session, 10 to 15 participants (the majority of whom were nurses, as well as nutritionists, laboratory workers, and maternity unit managers) gathered with two facilitators (either senior doctors or nurses working within the health zone management/coordination team or community health service providers). Documentation of the characteristics of participants of each session was incomplete in some cases, preventing an accurate analysis of the professional role, sex, and age of participants. (This was for two reasons – first, facilitators were often in a hurry to complete their tasks and secondl, they paid greater attention in their notes to the content issues that were raised and discussed).

The duration of the collaborative learning sessions was approximately two to three hours, to allow for adequate time for the stakeholders to immerse themselves in the session and actively contribute to the discussions. The sessions were held either at the central office of the health zone or at a health facility. Costs of transportation were covered for participants and facilitators, and refreshments were generally provided, but attendance was not financially incentivised.

Topics for the collaborative learning sessions were identified by the group using epidemiological data and health indicators to guide prioritisation. In most cases, the topic for the next session was selected by the group at the end of the current session. In addition, routine data analysis and brief situation analyses were conducted by some groups in order to highlight key issues affecting adolescents and young people, in the discussions. The topics of the sessions are presented in Table 1.

**Table 1.** Collaborative learning session topics

Health zone	Topics
**Diulu**	Introduction to sexual and reproductive health issues faced by adolescents (contraception, HIV/AIDS, gender-based violence, use of psychoactive substances, and unwanted early pregnancy)
	Clandestine/unsafe abortion
	Sexually transmitted infections among adolescents
	Early marriage and underlying factors
**Kalamu**	High rates of sexually transmitted infections among adolescents and girls
	High rates of early pregnancy among adolescent girls and young women
	Poor reception of adolescents and young people in health facilities
	The practice of respectful care for adolescents and young people (two sessions)
	Counselling for adolescents and young people who use psychoactive substances
	Counselling for the prevention of HIV among adolescents and young people
**Kansele**	HIV/AIDS among adolescents and young people
	Case discussion: suicide of a young girl who did not want a marriage imposed by her father
	Early marriage and divorce among adolescents and young people
	Early sexual activity among adolescents and young people
**Kintambo**	Ensuring confidentiality and privacy in the provision of health care services to adolescents and young people
	Making health services welcoming and friendly for adolescents and young people
	Early pregnancy among adolescent girls and young women
	The consequences of early pregnancy for adolescents and young people
	The promotion of contraception and family planning among adolescents and young women
	The prevention of sexually transmitted infections including HIV among adolescents and young people
	The prevention of infections among adolescents and young people
	Early sexual activity among adolescents and young people
**Makala**	Control of sexually transmitted infections among adolescents and young people
	HIV/AIDS prevention, treatment and care
	Promotion of the correct use of condoms
	Early pregnancy and unsafe abortion among adolescent girls and young women
	Sexual violence
	The prevention of early pregnancy among adolescent girls and young women
	Sexual intercourse and its consequences for adolescents and young people
**Nzaba**	The consumption of traditional alcohol among adolescents and young people
	Adapting support to adolescents (case discussions)

Each session addressed the topic at hand and factors influencing it, as well as appropriate approaches to address it in the local context. Key recommendations were developed, with discussion about how to implement them. Several session reports also included case discussions, which highlighted the complex SRH challenges faced by adolescents in the DRC. Issues raised included early unintended pregnancym unsafe abortionm coerced sex and gender-based violence, early marriage, sexually transmitted infections including HIV, social pressure for girls to maintain ‘virginity’, and myths and misconceptions about the health effects of masturbation.

### Costs

The total cost of the collaborative learning approach, from 2017 to 2019, including all the baseline training in ASRH for the health providers, training in the approach for the collaborative learning session facilitators, organization of the collaborative learning sessions themselves, and monitoring of these sessions was approximately USD 135,000.00. The majority of these costs relate to the initial training of participants and facilitators, technical support and monitoring missions performed by WHO, and printing and dissemination of materials. As this was a new approach in the context of DRC, it required initial investments and oversight to ensure that the model was designed and implemented with fidelity. The cost per session once the approach was being implemented was affordable in the local context, i.e. USD 30.00 per participant (see Tables 2–5).

**Table 2.** Training costs

Area of expenditure	Cost (USD)
Baseline training in ASRH for 50 health service providers, Kinshasa, 2017	20,241
Baseline training in ASRH for 50 health service providers, Kasaï Oriental and Mbuji-Mayi, 2018	27,358
Training on collaborative learning model for 24 collaborative learning session facilitators, 2018	*29,786*
**Total**	**77,385**

**Table 3.** Supervision and monitoring costs

Area of expenditure	Cost (USD)
Development of monitoring tools	961
Three supervision visits to identified health services per quarter, 2018	450
Technical supports and monitoring visits by WHO Country Office and Regional Office staff in 2018	13,440
Technical support and monitoring visits by WHO Country Office and Regional Office staff in 2019	13,740
**Total**	**28,591**

**Table 4.** Printing and dissemination costs

Area of expenditure	Cost (USD)
*Production of the Collaborative Learning Guide, 2018*	*1200*
Reprinting and dissemination of norms, standards and guidelines, 2019	9479
**Total**	**10,679**

**Table 5.** Cost per collaborative learning session by year (including transport, refreshments, and room hire fees)

Year	Number of sessions	Total cost (USD)	Cost per session (USD)
*2018*	*18*	*8400*	*467*
*2019*	*14*	*10,140*	*724*
**Total**	***32***	***18,540***	***579***

### Benefits

Review of the reports on the collaborative learning sessions suggests that areas were frequently identified where health service improvements were needed. Most of the collaborative learning groups developed recommendations for steps to address identified issues, suggesting that the sessions may have stimulated actions to improve the quality of health services provided by individual providers and at the facility level. These are related to three main areas:

1. Strengthening the ‘welcome’ offered to adolescents at health facilities, including by identifying and analysing challenges affecting adolescents and barriers to their access to health services; improving health service provider attitudes and motivation; and addressing other factors contributing to the delivery of non-judgemental, empathic and confidential health services that meet the needs and preferences of adolescents;
2. Addressing knowledge and skill gaps and sub-standard clinical care by health care providers;
3. Improving communication and collaboration between the health facility and adolescents together with their parents, by strengthening the provision of information and education on SRH issues, among other health issues such as mental health.

Regarding the first area, the most frequently proposed solution was additional training for health service providers on respectful provision of services to adolescents and the importance of addressing psychosocial factors affecting their health. It is likely that the collaborative learning sessions themselves contributed to improved health service provider attitudes and understanding of the health and broader issues faced by adolescents, although this was only implicitly recognised in session reports.

The second area was frequently addressed through additional capacity-building activities for health service providers, which, in some cases, were collaborative learning sessions (e.g. holding an education session on approaches to prevent early unintended pregnancy). In some instances, participants raised cases where adolescents were believed to have received sub-standard clinical care, which led to proposals on improving adherence to (or developing new) guidelines or protocols. In one example, a new procedure was developed to ensure that all adolescents presenting with an STI were offered a follow-up appointment; in another, a particular clinical team was declared responsible for ensuring that adolescents had access to contraceptive services, particularly post-partum and post-abortion contraception.

For the third area, in most instances, the proposed approach for delivering information and education to adolescents and their parents was not specified, but one proposed idea was to foster intergenerational dialogue by developing a ‘youth club’ to discuss SRH issues.

The information provided in the session reports was insufficient to assess whether recommendations arising from the collaborative learning sessions were successfully implemented or resulted in improved quality of health services.

Thematic analysis of the session and monitoring reports suggests that health service providers valued the collaborative learning approach and identified several benefits arising from it, including improved attitudes, knowledge and understanding of the health problems of adolescents and increased use of standards and guidelines, leading to improved quality of health services for adolescents. Participants noted that facilitators have been effective in encouraging the implementation of ideas raised during the sessions to improve services for adolescents. It was reported that in one health zone, some adolescents want to see only the health service providers who participated in the training and collaborative learning sessions. In another health zone, one health facility re-organised its services to better meet the needs of adolescentsfor example, by having a separate reception area for them and a private room for confidential discussions. Facilitators and health service providers reported that monitoring missions undertaken by the WHO Regional Office for Africa in collaboration with PNLS and PNSA were an important complement to the collaborative learning sessions to improve staff capacity, as well as the quality of services provided.

### Implementation challenges

Thematic analysis of programmatic documents as well as monitoring reports suggests that the greatest challenge in implementing the collaborative learning approach was its novelty in a setting in which the model of health service provider education has traditionally been hierarchy-conscious, formal and didactic and in which humanitarian and development projects are systematically incentivised. Learning and sharing in multi-disciplinary groups, where all participants are expected to contribute regardless of their status and seniority, was a new concept for many participants. This meant that time, discussion, and skillful facilitation were needed to establish a safe environment in which all session participants were comfortable and able to contribute equally to the discussions.

Logistical issues were also identified as a barrier to the successful roll-out of the collaborative learning approach. Monitoring reports noted that the delayed availability of the final version of the national collaborative learning guide at the level of health zones, and the absence of related national norms and standards, impeded the approach’s implementation. Additional information, education and communication supports were reported to be needed to strengthen and sustain the approach’s implementation. The absence of mechanisms to promote collaboration between health zones and to ensure the implementation of recommendations from the collaborative learning sessions was also noted. Finally, competing priorities and lack of certainty about the financial sustainability of the approach after the conclusion of the overarching programme were identified as risks to its continuation.

## Interpretation

This is the first study to explicitly focus on the use of collaborative learning in adolescent health services outside Europe, and the results suggest that the approach is both feasible and affordable in the local context. Our results also offer preliminary evidence of the acceptability of collaborative learning, although no incentives for participation were provided. This is important given that participation in health system improvement and other development projects has often been financially incentivised in the DRC and elsewhere, along with the novelty of such an informal and ongoing sharing and learning process in this context. Potential benefits of the collaborative learning approach include improved knowledge and skills among health service providers, strengthened ‘adolescent-friendliness’ of health services, and improved communication and trust between health service providers and adolescents.

Our findings align with the evidence from other quality improvement processes that include collaborative learning-type approaches. For example, the ‘client-oriented, provider-efficient’ (COPE) process, developed by EngenderHealth as a quality improvement tool for family planning services, has been implemented in over 45 countries (in different contexts and with different population groups including adolescents), and has been shown to improve health service provider performance in areas including respectful care, information provision, personal communication, privacy, and clinical skills [28]. This suggests that collaborative learning approaches may have the potential to address well-documented challenges in ensuring the quality and friendliness of SRH services for adolescents [29–31].

Indeed, the experience with collaborative learning in the DRC highlights the urgent need for a comprehensive approach to performance improvement that goes beyond training to improve health service provider knowledge, skills, attitudes, and motivation. Our findings suggest that health service providers have many questions and concerns about the health issues and needs of adolescents, and how best to meet them. This was highlighted by the case discussions, which dealt with complex issues such as coerced sex, gender-based violence, unsafe abortion, and social expectations about the sexual behaviour of adolescents, particularly girls. These concerns are inherently connected to deeply held beliefs and biases and social norms and raise complex ethical and legal dilemmas. This points to the importance of enabling health service providers to learn from and support each other through discussion and reflection, as well as the need for comprehensive, multi-sectoral approaches to address the wider social determinants of adolescents’ health. As noted by Lesco et al., the inclusion of sensitive themes like violence and abortion in collaborative learning sessions could contribute to changing the values and attitudes of health service providers on key issues [14].

Despite its clear feasibility and affordability, the collaborative learning approach implemented in the DRC faces challenges to its sustainability, including lack of clarity about continuous funding. Integrating collaborative learning into existing health service plans and budgets could help address this. Efforts to ensure national ownership of the programme, for example, by scaling up the approach to other adolescent health services across the country with support from the Ministry of Health, and careful ongoing monitoring and evaluation may also help to ensure the longevity of the approach. Further steps to strengthen the approach could include the development of mechanisms to promote collaboration between health zones and to ensure implementation of arising recommendations.

While our study suggests collaborative learning is feasible and acceptable within adolescent health services, it faces some limitations. First, the content and quality of the session reports varied widely, making it difficult to assess accurately the characteristics of the sessions and their participants and to synthesise qualitative findings. Secondly, we were only able to gather limited data related to the acceptability of the approach, knowledge about which would be very helpful in strengthening the approach in the DRC and adapting it for other contexts. Third, the short implementation period at the time of this analysis means that it is difficult to draw conclusions about the approach’s sustainability.

Further research is needed to examine the impact of collaborative learning approaches on health service provider knowledge and skills, attitudes and motivation, the quality of delivered health services, and the utilisation of and satisfaction with health services by adolescents. An evaluation to be completed in 2022 will explore the acceptability and effectiveness of the collaborative learning approach, including its impact on health service provider knowledge and skill, attitudes and motivation, and performance [32]. Further study is also needed to confirm the generalisability of our findings for other contexts and types of health services, and to confirm that the implementation of collaborative learning approaches can be sustained. In the long term, it would be useful to explore the potential of collaborative learning to strengthen the capacity of health services to meet the needs of adolescents most vulnerable to poor SRH outcomes, such as young key populations at risk of HIV.

## Conclusion

This study suggests that collaborative learning is a feasible and affordable approach to support health service providers to improve their performance in providing adolescent SRH services. It provides preliminary evidence for the acceptability of the approach and identifies possible benefits for addressing gaps in health service provider attitudes, knowledge and skills, and improving the adolescent-responsiveness of health services. Further research is needed to confirm our results in other settings and to examine the impact of collaborative learning on health service provider attitudes, knowledge, and skills, as well as the quality and usage and of health services and in the sustainability of this approach.

## Supplementary Material

Supplemental Material

## Paper context

A competent and empathic health worker is central to an adolescent friendly health service. Given this, there is a great deal of attention to building health workers’ abilities and attitudes. However, in most cases, the only approach used is training; furthermore, training is often done badly. There is a pressing need to improve the quality of training and to move away from off-site, one-off in-service training. This needs to be combined with other proven approaches – job descriptions grounded in quality standards, pocket/desk reference tools and supportive supervision – as well as opportunities to learn from and with each other – collaborative learning.
