# Supplementary material for: A collaborative learning approach to improving health worker performance in adolescent sexual and reproductive health service provision: a descriptive feasibility study in six health zones in the Democratic Republic of the Congo
Source: Glob Health Action. 2021 Oct 31;14(1):1985228. doi: 10.1080/16549716.2021.1985228 (PMC8567869; doi:10.1080/16549716.2021.1985228)
Supplement: Supplemental Material [file ZGHA_A_1985228_SM8084.docx]

**Logic model for the Project titled: Preventing HIV in adolescent girls and young women in the Democratic Republic of Congo**

| **Intervention support activities** | **Interventions to address the determinants** | **Determinants of adolescent behaviours** | **Adolescent behaviours most directly related to these health outcomes** | **Health outcomes** |
| --- | --- | --- | --- | --- |
| **Schools:**  Secure buy in from the Ministry of Education  Work with the Ministry of Education to (i) agree on the teacher training and student education materials to be used (ii) agree on the training and ongoing support mechanism to be put in place for school managers and teachers (iii) secure permission to set up listening centers (where students could report on threats of violence and violence) and girls’ corners (to give them a safe space) (v) set up referral links with health facilities (iv) identify the schools and health facilities  Work with Ministry of Education authorities to (i) orient school managers (ii) train and support teachers (iii) set up listening centers and girls’ corners (iv) set up linkages with health facilities  **Health facilities:**  Secure buy in from the Ministry of Health  Work with the Ministry of Health (i) agree on approach to be used to improve health worker performance – good quality training, desk reference tools, collaborative learning and supportive supervision (ii) to agree on actions to be taken to strengthen health system readiness and performance (iii) select the health facilities.  Work with the Ministry of Health authorities to (i) orient health facility managers (ii) train health workers (iii) set up a collaborative learning and supportive supervision programme (iv) make the infrastructure and process improvements agreed upon, to health facilities.  **Community:**  Identify an organization that could identify, train and support community activists in engaging community members and leaders in interactive exchanges (community conversations, drama and discussion groups) to challenge and change community understanding, norms and practices on ASRH including GBV.  **Society:**  Identify and engage an organization to implement a communication and advocacy strategy to engage community leaders, journalists, government officials and political leaders to promote a shared understanding of the SRH needs and problems of adolescents (with a particular focus on HIV and GBV) and to build a sense of urgency to address them. | **Schools:**  Educate adolescents about  SRH and about GBV, and about where/when and how to seek care  Empower adolescents to act with confidence to protect themselves e.g. by refusing unwanted sex, negotiating safe sex when wanted, and obtaining help for protection and seeking care  Refer adolescents to SRH/HIV/GBV services when needed  Refer adolescents to social and legal services  Make the school a safer place: (i) announce that GBV will not be tolerated (ii) establish systems for students to report if GBV occurs to them or to others (iii) establish listening centers and girls corners.  **Health facilities:**  Provide SRH/HIV services that adolescents need in a friendly and responsive manner.  Provide care that is subsidized/free of charge.  **Community:**  Engage parents/care givers to discuss SRH/HIV and GBV with their sons/daughters/wards and encourage them to take steps to protect themselves and/or seek help when needed.  Stimulate parents/care givers to advocate for increasing educational and employment opportunities for adolescents and young people, for legal and policy reform to liberalize access to SRH including GBV prevention and care interventions, and for greater attention to/investment in ASRH including GBV prevention and care programmers. | **Individual:**   - Feel able to challenge GBV as the prevailing norm. - Feel empowered to challenge the occurrence of GBV. - Feel that they can resist GBV. - Feel able to avoid/remove themselves from situations where there are exposed to GBV because of social or economic reasons. - Feel able to challenge the prevailing norm of early sexual initiation. - Well aware of the risks of unprotected sex, and about how to protect oneself. - Feel able to refuse unwanted sex. - Feel able to negotiate safe sex. - Do not feel compelled to have unprotected sex for financial reasons. - Know where they can seek care. - Are able to obtain free/subsidized care. - Feel that they have the right to seek care.   **Immediate environment:**   - There is growing community willingness to discuss and challenge norms and traditions that normalize (i) early sexual activity (ii) sexual activity without protection (iii) coerced sex (iv) sex in exchange for favours (v) early child bearing. - There is less social pressure for from peers and family to subscribe to these norms. - Discussion on sex, and sexual and reproductive health and GBV occur in homes, in schools and elsewhere in the community. - Health services are available, accessible and acceptable to adolescents. - Adolescents are able to get free/subsidized health services. - Stigma associated with experiencing GBV and seeking care when GBV occurs is declining. - Perpetrators of GBV are increasingly held to account. - There are initiatives to prevent GBV or to respond to it when it occurs in schools, elsewhere in the community and in health facilities. - Health workers and health services are required to, and trained and supported to respond effectively and with sensitivity to GBV.   **Wider Environment:**   - There are efforts to increase opportunities for study and paid work. - Laws and policies no longer restrict the provision of SRH information and services, - Programmes to promote the SRH of adolescents are better funded, and executed. - Programmes to prevent and respond to GBV are better funded and executed. | - Carrying out GBV - Not taking actions to avoid GBV/not seeking help to avoid GBV - Not seeking care and support when GBV occurs - Starting sexual activity early - Having unprotected sexual activity - Not seeking care to prevent and to respond to SRH problems (contraceptives including condoms, STI management, HIV testing and counselling, Pre and post exposure prophylaxis, and HIV care) | - HIV/STI, - Mortality and morbidity resulting from HIV/STI - Early pregnancy - Mortality and morbidity resulting from early pregnancy - GBV - Mortality and morbidity resulting from GBV |
